# Lung uptake of two SPECT markers identifies sensitivity to hyperoxia-induced acute respiratory distress syndrome in rats

**DOI:** 10.3389/fphys.2025.1648159

**Published:** 2025-09-12

**Authors:** Anne V. Clough, Pardis Taheri, Guru P. Sharma, Ming Zhao, Elizabeth R. Jacobs, Said H. Audi

**Affiliations:** ^1^ Department of Mathematical and Statistical Sciences, Marquette University, Milwaukee, WI, United States; ^2^ Joint Department of Biomedical Engineering, Medical College of Wisconsin, Marquette University, Milwaukee, WI, United States; ^3^ Research Service, Zablocki VA Medical Center, Milwaukee, WI, United States; ^4^ Department of Radiation Oncology, Medical College of Wisconsin, Milwaukee, WI, United States; ^5^ Department of Medicine (Cardiology), Feinberg School of Medicine, Northwestern University, Chicago, IL, United States; ^6^ Department of Medicine (Pulmonary), Medical College of Wisconsin, Milwaukee, WI, United States

**Keywords:** ARDS, molecular imaging, glutathione, HMPAO, single photon emission computedtomography (SPECT)

## Abstract

**Introduction:**

Exposure of adult rats to hyperoxia is a well-established model of human Acute Respiratory Distress Syndrome (ARDS). Although rats exposed to 100% O_2_ display clinical evidence of lung injury after ∼40 h and death by 72 h, rats exposed to 60% O_2_ for up to 7 days show little sign of injury. However, when subsequently exposed to hyperoxia, these pre-exposed rats become more susceptible to ARDS. The objective of this study is to evaluate the ability of imaging biomarkers to track this hyperoxia susceptibility and to elucidate underlying mechanisms.

**Methods:**

Sprague-Dawley rats were exposed to either room air (normoxia), >95% O_2_ for 24 h (hyperoxia), 60% O_2_ for 7 days (H-S), or H-S followed by 24 h of hyperoxia (H-S+24). Following i.v. injection of ^99m^Tc-duramycin (marker of cell death) and/or ^99m^Tc-hexamethylpropelyneamine oxime (^99m^Tc-HMPAO, marker of lung tissue redox status), *in vivo* scintigraphy images were acquired and lung uptake of these biomarkers was determined from the images.

**Results:**

^99m^Tc-HMPAO uptake was 84% greater in hyperoxic rats compared to normoxic controls. Uptake in H-S rats was 34% higher than normoxics, but with no change with subsequent exposure to hyperoxia (H-S+24). ^99m^Tc-duramycin uptake was 40% greater in hyperoxic rats than normoxics. Uptake in H-S rats was not different from normoxics but increased by 160% with H-S+24 in conjunction with enhanced hyperoxia susceptibility. ^99m^Tc-HMPAO and ^99m^Tc-duramycin uptake correlated with expression of 3-nitrotyrosine (oxidative stress) and cleaved-caspase 3 (cell death) measures acquired independently.

**Discussion:**

Overall, these results suggest the potential utility of ^99m^Tc-HMPAO and ^99m^Tc-duramycin imaging for identifying those hosts that are more, or less, susceptible to progression to severe ARDS at a time of mild symptoms of lung injury.

## Introduction

Acute Respiratory Distress Syndrome (ARDS) carries a mortality rate of approximately 40% due to lack of early detection tools, inability to identify patients at risk for progression, and limited therapies (Bellani et al., 2016; Fan et al., 2018; Levitt and Matthay, 2012, Fanelli et al., 2013) It is characterized by rapidly progressing hypoxic respiratory failure caused by direct and indirect sources including sepsis, pneumonia, systemic inflammation, trauma, and severe infections, including those caused by COVID-19 (Fan et al., 2018; Kallet and Matthay, 2013; Levitt and Matthay, 2012). Prior to COVID-19, the annual incidence in the U.S. of severe ARDS was ∼200,000 new cases (Levitt and Matthay, 2012; Ma et al., 2025).

Although high inspired oxygen (FiO_2_) ventilation, or hyperoxia, is not a common standalone cause of acute respiratory distress syndrome (ARDS), it is an unavoidable intervention in cases of severe ARDS (Fan et al., 2018). While often lifesaving, hyperoxia can itself contribute to lung injury, exacerbating pulmonary damage and increasing ICU mortality (Damiani et al., 2018; Girardis et al., 2016; Kallet and Matthay, 2013; Ligtenberg et al., 2013; Stolmeijer et al., 2013). Clinical studies have shown that liberal oxygen therapy in the ICU is associated with higher mortality among ARDS patients (Girardis et al., 2016; Chu et al., 2018), whereas conservative oxygen strategies are linked to reduced ICU mortality compared to conventional approaches (Girardis et al., 2016). Despite a wealth of cellular and molecular data regarding the pathogenesis of ARDS, the clinical course of patients with mild ARDS is still widely variable even for those with identical risk factors (Ware, 2005; Girardis et al., 2016; Bellani et al., 2016; Daenen et al., 2025). The Lung Injury Prevention Score (LIPS) can be used to identify patients at risk for severe ARDS (Gajic et al., 2011), however it has a low positive-predictive value. For instance, a LIPS cutoff of 4 is recommended as the criterion for implementing enhanced monitoring strategies. However, <7% of patients at that cutoff level actually progress to severe ARDS (Gajic et al., 2011). Individuals at elevated risk for oxygen-induced lung injury may be candidates for interventions such as tolerance of lower blood oxygenation (e.g., 86% saturation threshold), lower Hb thresholds for transfusions (due to the risk of transfusion reactions) or prone positioning. These therapies each carry risks of their own, and thus are not generally applied to all persons with ARDS. Thus, novel clinical means for stratifying the risk of severe ARDS in individual hosts with mild ARDS are needed.

Rat exposure to hyperoxia is a well-established model of human ARDS (Crapo et al., 1980; Kallet and Matthay, 2013). For most animal models, prolonged exposure to a 100% O_2_ environment is lethal due to acute lung injury (ALI) (Crapo and Tierney, 1974). Furthermore, when adult rats are exposed to 60% O_2_ for 7 days, they become more susceptible to the lethal effects of hyperoxia, as evidenced by a decrease in their subsequent survival time in 100% O_2_ (Hayatdavoudi et al., 1981). Conversely, when adult rats are preconditioned by exposure to 100% O_2_ for 2 days followed by a 24-h “rest period” in room air, they acquire tolerance of the otherwise lethal effects of hyperoxia (Frank et al., 1989; Crapo and Tierney, 1974). These unique models provide a platform for developing an approach to stratifying the risk of developing severe ARDS in animals with mild ARDS, and for elucidating the underlying mechanisms for such risk (Audi S. H. et al., 2012; Frank et al., 1989; Hayatdavoudi et al., 1981).

Oxidative stress, mitochondrial dysfunction, and inflammation are key pathways in the pathogenesis of ARDS, with the pulmonary capillary endothelium a primary and early target (Bannerman and Goldblum, 2003; Reiss, Uhlig, and Uhlig, 2012; Qiu et al., 2011; Xie et al., 2012; Chow et al., 2003; Herrero, Sanchez, and Lorente, 2018; Kellner et al., 2017). The pulmonary capillary endothelium, which has a large surface area and is in close contact with blood-borne compounds, can be targeted *in vivo* with single-photon emission computed tomography (SPECT) (Audi et al., 2016; Audi et al. 2022a; Audi et al. 2017; Audi S. H. et al., 2012; Clough et al., 2012; Audi et al., 2015). Previously, we demonstrated the utility of *in vivo* ^99m^Tc-hexamethylpropyleneamine oxime (HMPAO) and ^99m^Tc-duramycin imaging to detect oxidative stress and endothelial cell death, respectively, in rats exposed to hyperoxia (Audi et al., 2016; Audi et al. 2022a; Audi et al. 2017; Audi S. H. et al., 2012; Clough et al., 2012; Audi et al., 2015). Moreover, we recently demonstrated that *in vivo* ^99m^Tc-HMPAO and ^99m^Tc-duramycin imaging of pre-conditioned rats detects and predicts protection from ARDS when the rats are exposed to hyperoxia (Audi et al., 2022b). The results showed that a pattern of increasing lung uptake of ^99m^Tc-HMPAO and ^99m^Tc-duramycin correlates with advancing oxidative stress and cell death and worsening injury, whereas stable (or decreasing) ^99m^Tc-HMPAO and stable ^99m^Tc-duramycin reflects tolerance of hyperoxia. These outcomes suggest the potential utility of molecular imaging for identifying individual at-risk hosts that are more, or less, susceptible to progressing to more severe ARDS.

The objective of the current study is to evaluate the lung uptake of ^99m^Tc-HMPAO and ^99m^Tc-duramycin in rats with enhanced susceptibility for ARDS, before and after exposure to >95% O_2_. This will determine whether ^99m^Tc-HMPAO and ^99m^Tc-duramycin track this susceptibility. The activity of key cellular targets in H-S rats is also measured and correlated with injury and functional endpoints associated with ARDS to identify potential mechanisms of susceptibility that are tracked by ^99m^Tc-HMPAO and ^99m^Tc-duramycin.

## Materials and methods

### Materials

HMPAO (Ceretec®) was purchased in kit form from GE Healthcare (Arlington Heights, IL), and technetium-labeled macroaggregated albumin (^99m^Tc-MAA, particle sizes 20–40 μm) was purchased from Cardinal Health (Wauwatosa, WI). Duramycin (3,035 g/mol MW) kits were prepared as previously described (Audi et al., 2015; Clough et al., 2012). For western blots, antibodies for 3-nitrotyrosine (3-NT) were purchased from Abcam (primary: cat # ab61392) and Thermo Fisher (secondary: cat #31430), for mitochondrial complex I from Sigma-Aldrich (primary: cat # ABN302) and Thermo Fisher (secondary: cat #31430), and for β−actin from Bio-Rad (primary: cat #AHP2417) and Thermo Fisher (secondary: cat #31460). For immunohistochemistry antibodies for cleaved caspase 3 were purchased from Cell Signal Technology (primary cat#9661 and reagent cat #8114).

### Rat exposure to hyperoxia with and without preconditioning

All treatment protocols were approved by the Institutional Animal Care and Use Committees of the Clement J. Zablocki Veterans Administration Medical Center, Medical College of Wisconsin, and Marquette University.

For imaging and functional endpoint studies, male adult (10–12 weeks old) Sprague Dawley rats were obtained from Charles River, maintained on a 12 h day-night schedule, including during the study period, and experiments were performed during the day. They were randomized into four groups: 1.) Unconditioned: Rats (362.3 ± 40.1 (SD) g, n = 24) were housed in chambers with room air (normoxic controls). 2.) Unconditioned+24: Weight-matched rats (358.7 ± 23.9 g, n = 24) were housed adjacent to the room-air chambers in exposure chambers with >95% O_2_ for 24 h (h), as previously described (Audi et al., 2016). 3.) H-S: Another group of weight-matched rats (326.9 ± 24.6 g, n = 18) was housed in exposure chambers with 60% O_2_ for 7 days. 4.) H-S+24: The final group consisted of a subset of H-S rats (312.7 ± 12.1 g, n = 13) that were subsequently exposed to an additional 24 h of >95% O_2_. Twenty-four hours of subsequent exposure was chosen because the median time to death (LT50) for H-S rats in >95% O_2_ is 46 h, compared to 66 h for unconditioned rats (Hayatdavoudi et al., 1981). Since the time- or dose-response necessary to develop susceptibility to hyperoxia is not established in female rats, only male rats were used for this study.

For a randomly-selected subset of each group, the overall health of the rat prior to anesthetizing for the experimental protocols described below, was assigned a body score on a scale of 0–3 using the criteria shown in Table 1.

**TABLE 1:** Body scoring.

Body score	Body weight change	Physical appearance	Respiratory rate/Effort	Behavioral responses to external stimuli
0	Normal	Normal	Normal	Normal
1	<10% body weight loss	Lack of grooming, mildly rough coat	Minor increase in rate or effort	Minor depression, decreased activity
2	10%–15% body weight loss	Rough coat, nasal/ocular discharge (porphyrin accumulation)	Moderate increase in rate and effort	Moderately decreased activity (minimal exploratory activity in cage)
3	>20% weight loss	Very rough coat, abnormal posture (hunched), nasal/ocular discharge	Dyspnea (major increase in respir-atory effort/rate and/or open-mouth breathing)	Comatose or minimal (forced) response to tactile stimulation

### Lung wet-to-dry weight ratio

Heart and lungs were isolated as previously described (Audi et al., 2016). The lungs were then dissected free of the heart, trachea and mainstem bronchi and total lung wet weight was obtained. The left lung lobe was weighed and dried at 60 °C for 72 h for wet-to-dry weight ratio, and the remaining lung lobes were used for histological and western blot studies described below.

### Imaging studies

*In vivo* imaging studies described below were conducted on randomly selected subsets of rats from each group. The number of rats for each group were chosen to achieve a power ≥85% using power analysis (ANOVA power) based on previously published means and standard deviations of the lung uptake of ^99m^Tc-HMPAO and ^99m^Tc-duramycin (Audi et al., 2016; Audi et al., 2017; Audi S. H. et al., 2012; Clough et al., 2012; Audi et al., 2015; Audi et al., 2022b). As a control, in all imaging studies at least one normoxic unconditioned rat was imaged using the same prepared batch of radiopharmaceutical.

^99m^Tc-HMPAO and ^99m^Tc-duramycin were prepared as previously described (Audi et al., 2017; Audi et al., 2015; Clough et al., 2012), Rats were anesthetized (ketamine/xylazine: 80–100 mgkg^−1^/5–10 mgkg^−^1, i.p. or isoflurane: 0.5%–3%) and a tail vein was cannulated. The rat was then placed supine on a plexiglass plate (4 mm) positioned directly on the face of a parallel-hole collimator (hole diameter = 2 mm, depth = 25 mm) attached to a modular gamma camera (*Radiation Sensors, LLC*) for planar imaging (Audi et al., 2015; Audi et al., 2016).

**FIGURE 1:**
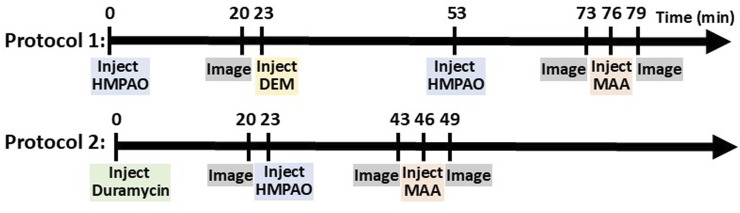
Diagram of imaging protocols showing the sequence of injections and imaging.

In one set of rats, an injection (37–56 MBq) of ^99m^Tc-HMPAO was administered via the tail vein catheter. ^99m^Tc-HMPAO reaches steady-state in the lung by 20 min post-injection, at which time five 30-s planar images were acquired (Audi et al., 2015; Audi et al., 2016). To investigate the role of the anti-oxidant glutathione (GSH) in the lung retention of ^99m^Tc-HMPAO, a random subset of rats were then treated with the GSH-depleting agent diethyl maleate (DEM, 1 g/kg body wt i.p.) (Audi S. H. et al., 2012). Thirty minutes after DEM treatment and without relocation of the rat, a second injection of ^99m^Tc-HMPAO was made and the animal reimaged 20 min later. This protocol is shown in Figure 1 as Protocol 1.

In another set of rats, an injection (37–56 MBq) of ^99m^Tc-duramycin was first administered via the venous catheter and images were acquired 20 min post-injection. Subsequently, ^99m^Tc-HMPAO was administered via the same venous catheter and the rats were re-imaged 20 min post-HMPAO injection (Figure 1, Protocol 2).

In all rats, a final injection of ^99m^Tc-MAA (37 MBq) was made via the same venous cannula and the rat reimaged. The ^99m^Tc-MAA injection provided a planar image in which the lung boundaries were clearly identified, since >95% of ^99m^Tc-MAA lodges in the lungs (Clough et al., 2012). After imaging, the rats were euthanized with an overdose of ketamine/xylazine (160–200 mg/kg: 10–20 mg/kg i.p.).

### Image analysis

The planar images were analyzed using MATLAB-based software developed in-house. The boundaries of the upper portion of the lungs were identified in the high-sensitivity ^99m^Tc-MAA images and manually outlined to construct a lung region of interest (ROI) free of liver contribution (Audi et al., 2016). The ^99m^Tc-MAA lung ROI mask was then superimposed on the biomarker image (^99m^Tc-HMPAO, ^99m^Tc-duramycin, or ^99m^Tc-HMPAO - ^99m^Tc-duramycin depending on the imaging protocol) yielding a lung biomarker ROI. No registration was required since the animal was maintained in the same location throughout the imaging study. Background regions in the upper forelimbs were also identified in the biomarker image to normalize lung activity for injected biomarker specific activity, dose, and decay (Audi et al., 2016; Audi et al., 2015). Mean counts/sec/pixel/injected dose within both the lung and forelimb-background ROIs were then determined. The ratio of the lung and background ROI signals averaged over the 5 × 30 s time interval, when the biomarker signal within the ROIs had reached steady state, was used as the measure of lung biomarker uptake (Audi et al., 2016; Audi et al., 2015).

### Histology

In a randomly selected subset of rats from each group, excised lungs were fixed after inflation in 10% neutral buffered formalin (Fisher Scientific, Pittsburg, PA) and embedded in paraffin. Whole-mount sections of lung were cut (4 μm thick) and stained with hematoxylin and eosin (H&E, Richard Allan, Kalamazoo, MI). Using NDP.view2 (Hamamatsu) to visualize the high-resolution images of the slides, two investigators, masked to the treatment groups, obtained six representative images from pre-selected areas of each lung on each slide, avoiding large vessels or airways. We used a 0–2 scoring system published by Matute-Bello et al. for neutrophil influx, edema, and thickness of the diffusion barrier, cardinal features of ARDS (Table 2) (Matute-Bello et al., 2011). Each image was scored independently by both investigators, and the six image scores per animal were averaged to yield a single score per feature. Inter-observer reliability was high, with intraclass correlation coefficients (ICCs) exceeding 0.95 for all three features. Final scores for each feature in each treatment group were then calculated by averaging the single scores from both investigators on all animals in that group.

**TABLE 2:** Lung histology scoring.

Histology score	Neutrophilic influx	Edema	Alveolar septum thickness
0	None to very rare	None to very rare	≤1.8 × control thickness
1	Perivascular or peribronchiolar only	Proteinacious material in >5% and <20% field	>1.8 and <2.5 × control thickness
2	intra-alveolar and widely distributed	Proteinacious material in >20% field	>2.5 × control thickness

Additional lung sections were immunostained with antibody to cleaved caspase 3 (CC3, a marker of caspase activation). Again, using jpeg images and ImageJ software, image thresholding was used to identify CC3-positive cells stained brown. Those cells were then colorized red for enhanced visualization in the images. The number of CC3 positive cells per image was counted by two investigators blinded to the treatment groups (with ICC = 0.91), and then averaged as described above for the histology images.

### Western blots

Western blot analysis was carried out as previously described on whole lung tissue homogenate to quantify the expression of 3-nitrotyrosine (3-NT), as an indicator of oxidative stress [for electrophoresis, protein used was 30 μg/lane (Audi et al., 2017)]. For complex I expression, the selected primary antibody recognizes NADH-ubiquinone oxidoreductase protein in complex I (Madhu et al., 2020). The protein is a 75 kDa subunit of complex I that catalyzes electron transfer from NADH. Approximately 50 mg of lung tissue was homogenized in 500 μL of Radio-Immunoprecipitation Assay (RIPA) buffer. After centrifugation for 10 min at 10,000 g to remove debris, supernatants were denatured at 480 °C for 5 min. 30 μg protein/lane were loaded for electrophoresis. The primary complex I antibodies were diluted 1:250 in non-fat dry milk and Tris-buffered saline and Tween (TBST). After electrophoretic separation and washing, matched secondary antibodies were applied, and blots visualized using ECL Plus detection reagent. Complex I blots were cut at 50 kDa. The higher molecular weight band was probed for complex I (75 kDa) and the lower molecular weight band for β-actin (42 kDa). For a given lane and protein, the protein density was estimated by ImageJ software and was normalized to the corresponding density for β-actin (42 kDa). The resulting value is referred to as “relative protein.”

### Statistical analysis

Statistical evaluation of data was carried out using GraphPad Prism v. 10.2.3 (Dotmatics Inc., Boston, MA). The level of statistical significance was set at 0.05 for all tests. Group results are expressed as mean ± SEM, unless noted otherwise. One of our primary objectives was to elucidate the differential effect of 24 h of hyperoxia on unconditioned versus preconditioned (H-S) rats. Thus, unless otherwise stated, we evaluated differences between two corresponding groups of rats (e.g., unconditioned vs. unconditioned+24 or H-S vs. H-S+24) using an unpaired two-tailed *t*-test for data passing the Kolmogorov-Smirnov normality test, or the nonparametric equivalent Mann-Whitney U test when data were not normally distributed.

## Results

### Body weight, lung wet/dry weight ratios, body score

Table 3 shows that rats exposed to 60% O_2_ for 7 days (H-S) gained body weight at a rate consistent with that for unconditioned rats (Gan et al., 2011). Unconditioned and H-S rats exposed to hyperoxia (100% O_2_ for 24 h) had no change in body weight. Exposure to hyperoxia or to H-S resulted in no changes in lung wet weight or wet/dry weight ratio compared to unconditioned (Table 4). Exposure to hyperoxia (H-S+24) following preconditioning (H-S) resulted in increases in both wet weight (43%) and weight/dry weight ratio (13%), and in significantly increased (worsened) body scores compared to H-S.

**TABLE 3:** Rat body weights.

Body weight, g	Unconditioned + 24 (n = 24)	H-S (n = 18)	H-S+24 (n = 13)
Pre-exposure	358.7 ± 4.9	326.9 ± 5.8	366.5 ± 3.5
Post-exposure	357.1 ± 4.3	381.2 ± 6.6*	361.4 ± 3.6
% change	−0.4 ± 0.4	16.7 ± 0.6	−1.4 ± 0.1

Values are mean ± SEM; n = number of rats. Unconditioned+24: exposure to >95% O_2_ for 24 h; H-S: exposure to 60% O_2_ for 7 days; H-S+24, H-S followed by exposure to >95% O_2_ for 24 h. For H-S+24 rats, pre-exposure body weight is that following exposure to 60% O_2_ for 7 days. (*) Significantly different from pre-exposure body weight, paired *t*-test, *p* < 0.001.

**TABLE 4:** Injury endpoints and lung histology scores.

Condition	Unconditioned	Unconditioned +24	H-S	H-S+24
Wet weight, g	1.32 ± 0.07 (n = 11)	1.39 ± 0.02 (n = 12)	1.34 ± 0.06 (n = 5)	1.92 ± 0.26^#^ (n = 8, *p* = 0.006)
Wet-to-dry weight ratio	5.27 ± 0.23 (n = 10)	4.99 ± 0.05 (n = 12)	5.08 ± 0.04 (n = 5)	5.76 ± 0.35^#^ (n = 8, *p* = 0.045)
Neutrophilic influx	0 ± 0 (n = 5)	0.18 ± 0.05 * (n = 8, *p* = 0.020)	0.83 ± 0.07 (n = 5)	1.67 ± 0.08^#^ (n = 8, *p* = 0.004)
Edema	0 ± 0 (n = 5)	0.24 ± 0.06 * (n = 8, *p* = 0.013)	0.67 ± 0.08 (n = 5)	0.95 ± 0.17^#^ (n = 6, *p* = 0.028)
Alveolar septum thickness	0 ± 0 (n = 5)	0.21 ± 0.04* (n = 8, *p* = 0.015)	0.50 ± 0.04 (n = 5)	1.33 ± 0.06^#^ (n = 6, *p* = 0.002)
Body Score	0 ± 0 (n = 24)	0.28 ± 0.14 * (n = 18, *p* = 0.004)	0 ± 0 (n = 14)	1.69 ± 0.85^#^ (n = 13, *p* < 0.001)

Values are mean ± SEM, with number of lungs (n) in parentheses. Unconditioned+24: exposure to >95% O_2_ for 24 h; H-S: exposure to 60% O_2_ for 7 days; H-S+24, H-S followed by exposure to >95% O_2_ for 24 h. All tests were done with Mann Whitney U test comparing (*) unconditioned to unconditioned+24 or (#) H-S to H-S+24.

### Imaging

**FIGURE 2:**
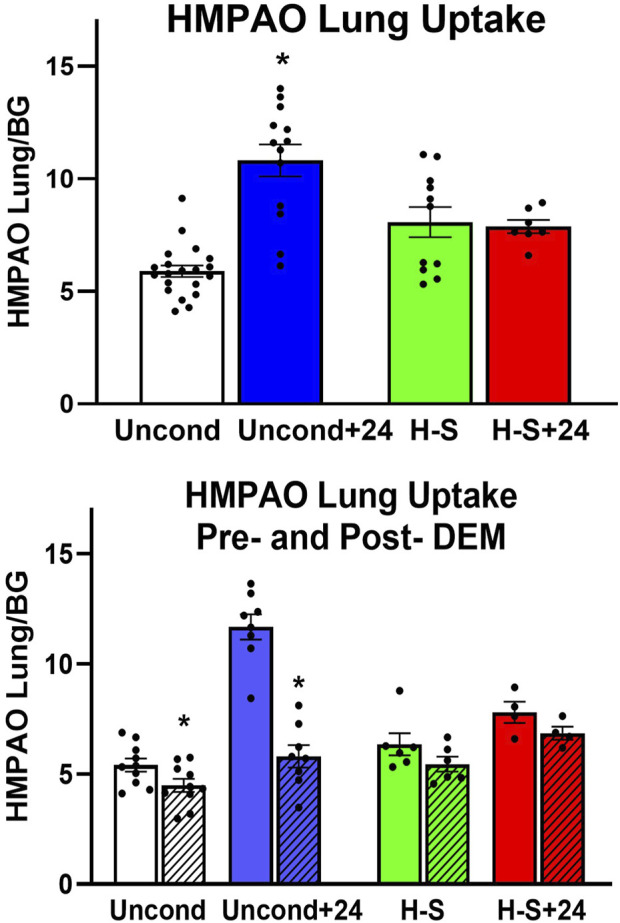
Top: Steady-state lung uptake [lung-to-background (BG) ratio] of ^99m^Tc-HMPAO. Uncond: Unconditioned normoxic controls (*n* = 20). Uncond+24: exposure to >95% O_2_ for 24 h (*n* = 13). H-S: exposure to 60% O_2_ for 7 days (*n* = 11). H-S+24: preexposure to H-S followed by exposure to >95% O_2_ for 24 h (*n* = 7). (*) Statistical difference between unconditioned and unconditioned +24 h of hyperoxia, unpaired *t*-test, *p* < 0.001. Bottom: Steady-state lung uptake of ^99m^Tc-HMPAO pre- (*solid bars*) and post- (hashed bars) administration of DEM (diethyl maleate) in the same rat. (*) Statistical difference between pre-DEM and post-DEM, paired *t*-test, *p* = 0.026 for unconditioned and *p* < 0.001 for unconditioned + 24 h of hyperoxia. The number of rats for unconditioned, unconditioned+24, H-S, and H-S+24 is *n* = 10, 8, 6, 4. Values are mean ± SEM.

Lung uptake of ^99m^Tc-HMPAO and ^99m^Tc-duramycin was quantified from the biomarker images obtained from the four groups of rats. Figure 2 (top) shows lung uptake (the ratio of lung-to-forearm background signal at steady-state) of ^99m^Tc-HMPAO in unconditioned+24 rats was 84% greater than in unconditioned rats. However, ^99m^Tc-HMPAO uptake was not statistically different between the preconditioned (H-S) and preconditioned with subsequent 24 h exposure to hyperoxia (H-S+24).

We investigated the role of GSH in ^99m^Tc-HMPAO lung uptake by imaging a subset of rats before and after treatment with DEM. A paired *t*-test was used to evaluate differences pre- and post- DEM for a given group. Figure 2 (bottom) shows that DEM treatment resulted in significantly reduced uptake in the unconditioned (15%) and unconditioned+24 (48%) groups, with a 12% but not statistically significant reduction in both the H-S and H-S+24 groups. These reductions are consistent with a role for GSH in the uptake of ^99m^Tc-HMPAO (Audi et al., 2016; Audi et al., 2017; Audi S. H. et al., 2012; Audi et al., 2022b; Clough et al., 2012). The reduction in ^99m^Tc-HMPAO uptake with DEM in unconditioned+24 (48%) was a greater fraction than that of unconditioned rats (15%). These data also indicate that lung uptake of ^99m^Tc-HMPAO in unconditioned+24 after DEM administration (blue hashed bar) was about the same as uptake in the unconditioned prior to DEM (white bar). This observation suggests that most of the increased uptake of ^99m^Tc-HMPAO in response to 24 h of hyperoxia (blue versus white bar) was a result of increased GSH content. Although the changes are smaller when comparing H-S to H-S+24, DEM again resulted in H-S+24 ^99m^Tc-HMPAO lung uptake (red hashed bar) about the same as that measured in H-S rats (green bar).

**FIGURE 3:**
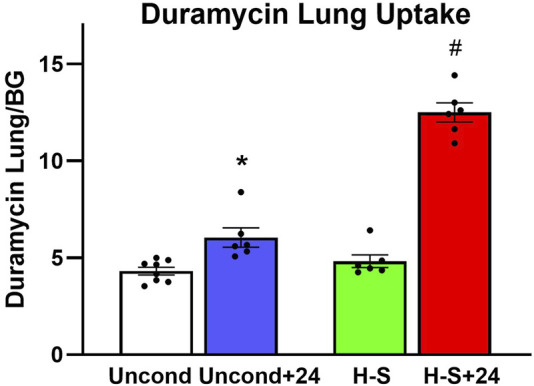
Steady-state lung uptake [lung-to-background (BG) ratio] of ^99m^Tc-duramycin. (*) Statistical difference between unconditioned and unconditioned + 24 h of hyperoxia, unpaired *t*-test, *p* = 0.004. (#) Statistical difference between H-S and H-S+24, unpaired *t*-test, *p* < 0.001. The number of rats for unconditioned, unconditioned+24, H-S, and H-S+24 is *n* = 8, 6, 6, 6. Values are mean ± SEM.

Figure 3 shows the results of ^99m^Tc-duramycin imaging. ^99m^Tc-duramycin uptake in lungs of unconditioned+24 rats was 40% greater than uptake in unconditioned normoxic rats. However, uptake in H-S+24 rats was 160% greater than H-S rats, associated with enhanced hyperoxia susceptibility/sensitivity.

### Histology

**FIGURE 4:**
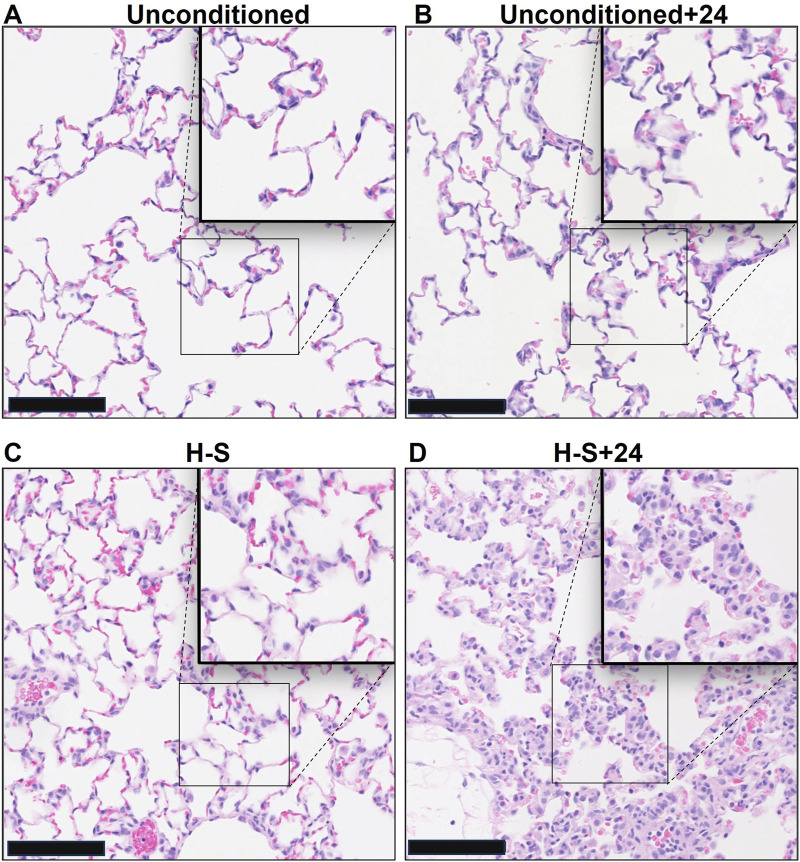
Representative unconditioned **(A)**, unconditioned+24 **(B)**, H-S **(C)**, and H-S+24 **(D)** rat lung slices stained with hematoxylin and eosin (H&E). Scale bar is 100 microns.

Images of representative lung sections stained with H&E appear in Figure 4. Figure 4A shows the lacy architecture, single-cell alveolar septum thickness and no evidence of neutrophil infiltration or edema, typical of normoxic unconditioned lungs. Figure 4B shows little histological evidence of injury in unconditioned lungs after just 24 h of hyperoxia (unconditioned+24). Figure 4C from a H-S rat lung shows some thickening of the alveolar wall and neutrophil infiltration. Figure 4D from an H-S+24 rat shows significant thickening of the alveolar wall with many inflammatory cells concentrated in the perivascular space, along with proteinaceous material in perivascular and intra-alveolar spaces (non-cellular pink staining). Mean injury scores from the images (Table 4) show small, although statistically significant, differences between lungs from unconditioned versus unconditioned+24 rats. However neutrophil, edema, and alveolar septum thickness injury scores in lungs from H-S+24 rats were all significantly higher than those of H-S rats, and larger than the differences between unconditioned and unconditioned +24 rats.

### Cleaved caspase 3 (CC3)

**FIGURE 5:**
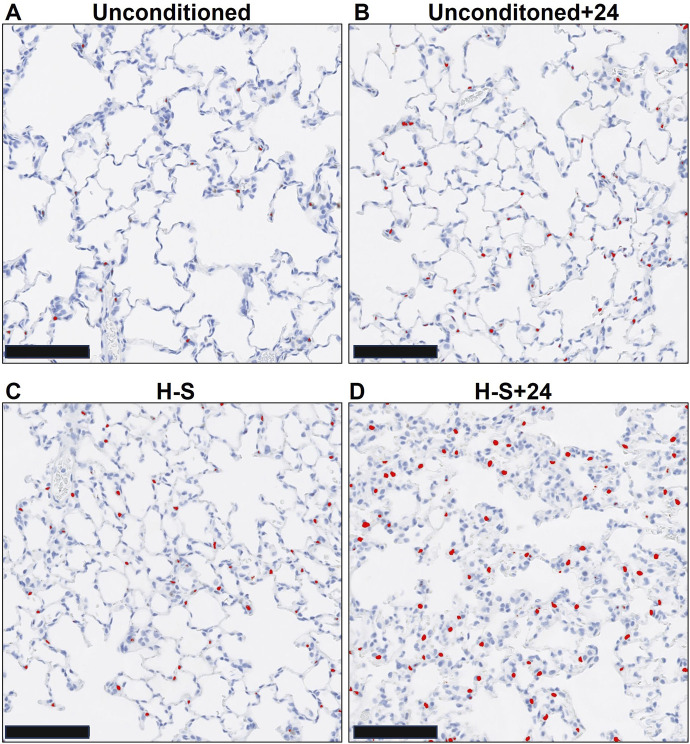
Representative unconditioned **(A)**, unconditioned+24 **(B)**, H-S **(C)**, and H-S+24 **(D)** rat lung slices stained with antibodies to cleaved caspase 3 (CC3) as a marker of apoptotic cells. CC3-positive cells are colorized red. Scale bar is 100 microns.

**FIGURE 6:**
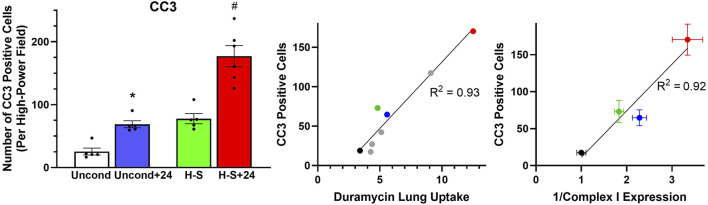
Left: Number of CC3-positive cells per high-power field. (*) Statistical difference between unconditioned and unconditioned + 24 h of hyperoxia, unpaired *t*-test, *p* = 0.008. (#) Statistical difference between H-S and H-S+24, Mann Whitney U test, *p* = 0.001. The number of rats for unconditioned, unconditioned+24, H-S, and H-S+24 is *n* = 5, 5, 5, 6. Center: Correlation between CC3-positive cells and ^99m^Tc-duramycin lung uptake in unconditioned (black), unconditioned+24 (blue), H-S (green) and H-S+24 (red) rats from this study, as well as other previously published data [gray (Audi et al., 2022b)]. Right: Relationship between CC3-positive cells and the reciprocal of Complex I expression (data in Figure 8 right below) from unconditioned (black), unconditioned+24 (blue), H-S (green) and H-S+24 (red) rat lungs. Values are mean ± SEM.

Figure 5 shows representative images of lung slices immunostained with antibody to CC3 as a marker of apoptotic cells. Scattered apoptotic cells are visible in unconditioned image (panel A), somewhat larger numbers are evident in unconditioned+24 (B) and H-S conditions (C), and a large number are present in the H-S+24 slice. Figure 6 (left) shows quantification of the number of CC3-positive cells per high-power field. The mean number in the unconditioned+24, H-S, and H-S+24 slices was ∼170%, 200% and 620% higher, respectively, than unconditioned (controls). Also, H-S+24 was 140% higher than H-S, consistent with duramycin imaging (Figure 3), providing additional evidence of hyperoxia sensitivity. Figure 6 (right) demonstrates that there is high correlation between lung uptake of ^99m^Tc-duramycin (Figure 3) and the number of CC3 positive cells (Figure 6, left, colored symbols), and that results are consistent with ^99m^Tc-duramycin imaging and CC3 measures obtained from rats exposed to different hyperoxia injury models (gray circles) (Audi et al., 2022b).

### Western blots

**FIGURE 7:**
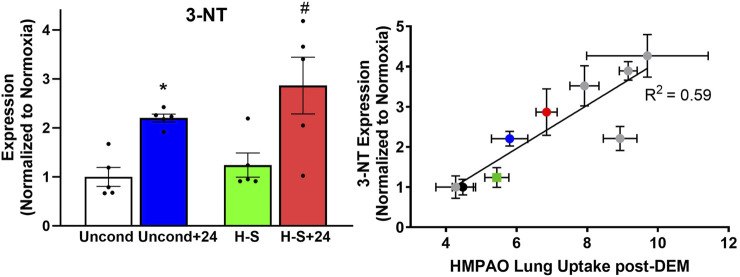
Left: Lung 3-NT protein expression (normalized to expression of β-actin) relative to unconditioned control value. (*) Statistical difference between unconditioned and unconditioned +24 h of hyperoxia, unpaired *t-*test, *p* < 0.001. (#) Statistical difference between H-S and H-S+24, unpaired *t-*test, *p* = 0.003. *n* = 5 rats for each group. Right: Correlation between lung 3-NT expression and ^99m^Tc-HMPAO lung uptake following DEM administration in unconditioned (black), unconditioned+24 (blue), H-S (green) and H-S+24 (red) rats from this study, as well as other previously published data (gray) (Audi et al., 2022b). Values are mean ± SEM.

We proceeded to assess the expression of 3-NT, a marker of oxidative injury, in the four groups using western blot analysis, with representative blots shown in Supplementary Figure S1. Quantification results shown in Figure 7 (left) indicate that 3-NT expression is 120% greater in unconditioned+24 lungs compared to unconditioned controls, and 130% greater in H-S+24 lungs than in H-S lungs. Figure 7 (right) shows good correlation between 3-NT expression and lung uptake of ^99m^Tc-HMPAO post DEM administration to deplete glutathione. This graph depicts new data from the current study using the colored symbols, while data from previous studies under different experimental hyperoxic conditions are shown by the gray circles (Audi et al., 2022b).

**FIGURE 8:**
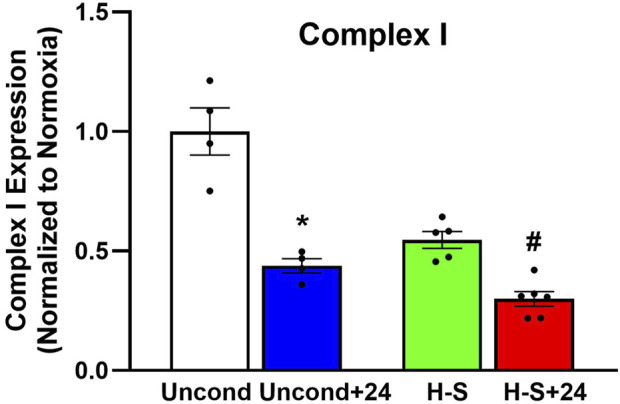
Lung mitochondrial complex I expression (normalized to expression of β-actin) relative to unconditioned control. (*) Statistical difference between unconditioned and unconditioned + 24 h of hyperoxia, Mann-Whitney U test, *p* = 0.029. (#) Statistical difference between H-S and H-S+24, Mann-Whitney U test, *p* = 0.004. The number of rats for unconditioned, unconditioned+24, H-S, and H-S+24 is *n* = 4, 4, 5, 6. Values are mean ± SEM.

To assess the role of mitochondrial dysfunction in rat susceptibility to hyperoxia, we also evaluated the expression of mitochondrial complex I in lungs using western blots. Figure 8 shows that Complex I expression was 44% lower in unconditioned+24 lungs compared to unconditioned controls. and 45% lower in H-S+24 relative to H-S, which in turn was 70% lower than unconditioned.

## Discussion

Consistent with this pre-exposure protocol, our results show that rats exposed to 60% oxygen for 7 days (H-S) become susceptible to hyperoxia-induced lung injury (Hayatdavoudi et al., 1981). This is evidenced by the relatively large increases in lung wet weight, histologic measures of injury, CC3, body scores, and lung tissue expression of 3-NT, after just 24 h of hyperoxia exposure (H-S+24). For these endpoints, the change in signal between H-S and H-S+24 was significantly larger than the change between unconditioned and unconditioned+24. For instance, Table 4 shows that unconditioned rats exhibited relatively small changes in histological markers of lung injury, including neutrophilic influx and edema, after 24 h of hyperoxia. In contrast, H-S rats displayed a larger increase in these markers following the same 24 h exposure period (H-S+24). Previously we showed that hyperoxia-tolerant (H-T) rats displayed smaller increases in lung injury endpoints (including those reported in the present study) than unconditioned rats following exposure to hyperoxia for 60 h (Table 5) (Audi et al., 2022b).

**TABLE 5:** Injury endpoints in rat models of hyperoxia.

Condition	Unconditioned	Unconditioned +24	Unconditioned +60	H-S	H-S+24	H-T	H-T+60
Lung wet weight, g	1.32 ± 0.07 (11)	1.39 ± 0.02 (12)	2.68 ± 0.17 (21)	1.34 ± 0.06 (5)	1.92 ± 0.26 (8)	1.60 ± 0.10 (10)	2.18 ± 0.06 (18)
Lung wet-to-dry weight ratio	5.27 ± 0.23 (10)	4.99 ± 0.05 (12)	6.36 ± 0.02 (12)	5.08 ± 0.04 (5)	5.76 ± 0.35 (8)	4.99 ± 0.07 (8)	5.75 ± 0.11 (21)
Neutrophilic influx	0 ± 0 (5)	0.18 ± 0.05 (8)	1.44 ± 0.13 (6)	0.83 ± 0.07 (5)	1.67 ± 0.08 (6)	0.21 ± 0.04 (4)	0.89 ± 0.03 (11)
Edema	0 ± 0 (5)	0.24 ± 0.06 (8)	0.81 ± 0.10 (6)	0.67 ± 0.08 (5)	0.95 ± 0.17 (6)	0.17 ± 0.07 (4)	0.12 ± 0.04 (11)
Alveolar septum thickness	0 ± 0 (5)	0.21 ± 0.04 (8)	1.03 ± 0.11 (6)	0.50 ± 0.04 (5)	1.33 ± 0.06 (6)	0.38 ± 0.08 (4)	0.68 ± 0.10 (11)
Body Score	0 ± 0 (24)	0.28 ± 0.14 (18)	3.0 + 0 (8)	0 ± 0 (14)	1.69 ± 0.85 (13)	0 + 0 (7)	1.64 ± 0.17 (22)
CC3	25.6 ± 4.9 (5)	68.8 ± 5.0 (5)	117.4 ± 3.4 (4)	77.8 ± 7.2 (5)	183.7 ± 15.4 (6)	27.2 ± 0.6 (4)	42.4 ± 0.5 (4)
3-NT	1.0 ± 0.13 (6)	2.20 ± 0.08 (5)	3.89 ± 0.23 (4)	1.29 ± 0.25 (5)	2.86 ± 0.58 (5)	3.52 ± 0.5 (4)	4.27 ± 0.53 ()
*K*_*f*_	2.40 ± 0.26 (6)	2.4 ± 0.68 (6)	7.01 ± 0.90 (6)	6.36 ± 0.87 (4)	NA	1.56 ± 0.39 (4)	2.88 ± 0.65 (5)

Values are mean ± SEM, with number of lungs in parentheses. Unconditioned+24: exposure to 100% O_2_ for 24 h; H-S: exposure to 60% O_2_ for 7 days; H-S+24, H-S followed by exposure to 100% O_2_ for 24 h; H-T: exposure to 100% O_2_ for 48 h followed by 24 h of room air; H-T+60: H-T followed by exposure to 100% O_2_ for 60 h. Data for unconditioned+60, H-T, and H-T+60 are from Audi et al. (2022b).

Imaging results in Figures 2, 3 indicate that susceptibility to hyperoxia is marked by a lack of change in ^99m^Tc-HMPAO uptake and a significant increase in ^99m^Tc-duramycin uptake in lungs of H-S rats exposed to hyperoxia for 24 h (H-S+24). In contrast, unconditioned rats subjected to 24 h of hyperoxia show a substantial increase in ^99m^Tc-HMPAO uptake, along with only a modest increase in ^99m^Tc-duramycin uptake.

Normobaric hyperoxic exposure causes a dose-dependent oxidative injury consistent with ARDS in rats and humans (Fisher and Beers, 2008). Rodent hyperoxia is a good model of human ALI, and is particularly relevant because of the two-hit hypothesis of lung injury (Hoegl et al., 2018). To this point, hyperoxia *alone* is not a common cause of ARDS, but its use is unavoidable in patients with this condition (Fan et al., 2018).

While lifesaving, ventilation with gases with high fractions of oxygen (FiO_2_) itself leads to injury amplifying lung damage (Gajic et al., 2011; Chen and Ware, 2015; Santos et al., 2016; Villar et al., 2016; Fisher and Beers, 2008; Kallet and Matthay, 2013; Stolmeijer et al., 2013; Ligtenberg et al., 2013). Rats exposed to hyperoxia develop increased lung wet weights, histological and imaging features of ARDS, including higher edema scores that translate to roentgenographic infiltrates in humans (Table 4), and increased gradients to oxygen exchange (Audi et al., 2016). Pre-exposures or treatments render rats more or less susceptible to lung injury (Frank et al., 1989; Taheri et al., 2024; Audi et al., 2022b; Hayatdavoudi et al., 1981). Thus, our injury model is highly relevant to clinical ARDS, and is optimally suited to testing the potential of lung imaging to reflect either evolving oxidative states and damage or disease regression.

**FIGURE 9:**
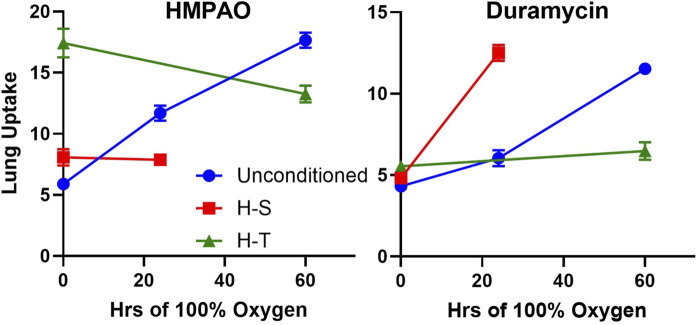
Lung uptake of ^99m^Tc-HMPAO (left) and ^99m^Tc-duramycin (right) in unconditioned (blue circles), H-S (hyperoxia-sensitive, red squares), and H-T (hyperoxia-tolerant, green triangles [Audi et al., 2022b)] exposed to up to 60 h of >95% O_2_ hyperoxia.

Previously, we demonstrated the ability of ^99m^Tc-HMPAO and ^99m^Tc-duramcyin to track hyperoxia tolerance in a rat model (Audi et al., 2022b). Specifically, we found that hyperoxia-tolerant (H-T) rats exhibited either stable or decreased ^99m^Tc-HMPAO lung uptake in response to 60 h of hyperoxia exposure, while ^99m^Tc-duramycin lung uptake remained unchanged. Conversely, unconditioned rats displayed progressive increases in both ^99m^Tc-HMPAO and ^99m^Tc-duramycin lung uptake over the 60-h exposure (Audi et al., 2022b). These findings alongside those from the current study are depicted in Figure 9, where these distinctive trends are observed in response to hyperoxia. Thus, molecular imaging with these biomarkers at two different time points could be a promising tool to stratify susceptibility of subjects to lung injury (as measured by the non-imaging invasive end points in this study not possible in humans) when exposed to hyperoxia.

The findings of this study also build upon and extend our previous work, where we demonstrated the utility of ^99m^Tc-HMPAO imaging for early detection of lung injury, even in the absence of clinically significant symptoms, following exposure to hyperoxia or low-dose lipopolysaccharide (LPS) (Audi et al., 2016). That work also showed its capability to track the progression of hyperoxia-induced acute lung injury (ALI) and the reversibility of LPS-induced ALI. Furthermore, we established that both ^99m^Tc-HMPAO and ^99m^Tc-duramycin can detect the protective effects of inhaled hydrogen therapy against hyperoxia-induced ALI (Audi et al., 2017).

### ^99m^Tc-HMPAO lung uptake

^99m^Tc-HMPAO was originally developed as a brain perfusion agent but its uptake and retention in other tissues has been shown to serve as a marker of tissue redox state (Neirinckx et al., 1988; Jacquier-Sarlin, Polla, and Slosman, 1996). Uptake of its oxidized (lipophilic) form from the circulation is dependent on its rate of diffusion across the plasma membrane (Andersen et al., 1988; Neirinckx et al., 1988; Clough et al., 2019). ^99m^Tc-HMPAO reduction and thus its cellular retention, has been shown to be strongly dependent on the oxidoreductive state of the tissue including intracellular glutathione (GSH) content and other factors involving mitochondrial function (Andersen et al., 1988; Neirinckx et al., 1988; Clough et al., 2019; Audi S. H. et al., 2012).

Previously, we showed that ^99m^Tc-HMPAO lung uptake can be separated into GSH- dependent and independent components by assessing its lung uptake prior and post administration of the GSH depleting agent DEM (Audi et al., 2016; Audi et al., 2017; Audi S. H. et al., 2012; Audi et al., 2022b; Clough et al., 2012). The portion of measured ^99m^Tc-HMPAO uptake that is DEM-insensitive, i.e., GSH-independent, is shown in Figure 2 (bottom) by the hashed bars (post-DEM), while the difference between the solid bars and the hashed bars represents the DEM-sensitive (GSH-dependent) component. The increased ^99m^Tc-HMPAO lung uptake in unconditioned rats following exposure to hyperoxia for 24 h [Figure 2 (top)] reflects the response of those lung cells accessible to ^99m^Tc-HMPAO to hyperoxia-induced oxidative stress. Figure 2 (bottom) shows that most of this increase was GSH-dependent in that following DEM administration unconditioned+24 lung uptake (blue hashed bar) was similar to unconditioned lung uptake (white bar). This is consistent with the ability of lung cells of unconditioned rats to respond to oxidative stress by increasing their antioxidant capacity, and with results from a previous study that showed that exposure of unconditioned rats to hyperoxia for 24 h increased lung GSH tissue content by ∼17% (Audi et al., 2016).

For H-S rats, Figure 2 (top) shows that there was no significant change in ^99m^Tc-HMPAO lung uptake following exposure to hyperoxia for 24 h, and that lung uptake of ^99m^Tc-HMPAO in both H-S and HS+24 rats was predominantly DEM-insensitive. These results are consistent with the lack of response from lung cells accessible to ^99m^Tc-HMPAO in both H-S and H-S+24 rats to oxidative stress, including change in their antioxidant capacity. This may contribute to the hyperoxia susceptibility of H-S rats. Previously we reported an increase (∼19%) in lung tissue GSH content of H-S rats compared to that in unconditioned rats (Audi S. H. et al., 2012), which as stated above, could be in cells not accessible to ^99m^Tc-HMPAO.

In our previous work investigating hyperoxia *tolerance*, we reported much higher ^99m^Tc-HMPAO lung uptake in H-T rats than in unconditioned rats, with most of the increase being DEM-sensitive (Audi et al., 2022b). We also reported ∼44% higher GSH content in lung tissue of H-T versus unconditioned rats (Audi et al., 2022b; Audi S. H. et al., 2012). These results are consistent with a higher antioxidant capacity in lung cells accessible to ^99m^Tc-HMPAO in lungs of H-T compared to unconditioned rats. Taken together, these studies provide strong evidence that rat tolerance or susceptibility to hyperoxia ALI is associated with its antioxidant capacity and/or the ability of the organism to rapidly increase its lung antioxidant enzymes in response to hyperoxia exposure as measured by the DEM-sensitive fraction of ^99m^Tc-HMPAO lung uptake.

Results from this study shown in Figure 2 (bottom) indicate that the DEM-insensitive (GSH-independent) component of ^99m^Tc-HMPAO lung uptake was unchanged following hyperoxia exposure in both the unconditioned (white hashed vs. blue hashed bars) and H-S rats (green hashed versus red hashed bars). Previous studies have suggested several GSH-independent factors that could contribute to ^99m^Tc-HMPAO tissue retention, including vascular permeability, mitochondrial dysfunction, and endothelial amine metabolism dysfunction (Ahn et al., 1994; Gardner et al., 2008; Kuo, Yang, and Chen, 2005; Shih et al., 1991; Suga et al., 1994; Audi et al., 2022b; Taheri et al., 2024). We have previously demonstrated that exposure of unconditioned rats to hyperoxia for 24 h had no effect on the pulmonary vascular endothelium filtration coefficient (*K*_*f*_) (Audi et al., 2016), which is consistent with the histological edema score in the present study (Table 4), and suggests that increased vascular permeability is not a strong contributor to the increased DEM-insensitive ^99m^Tc-HMPAO tissue retention reported here.

**FIGURE 10:**
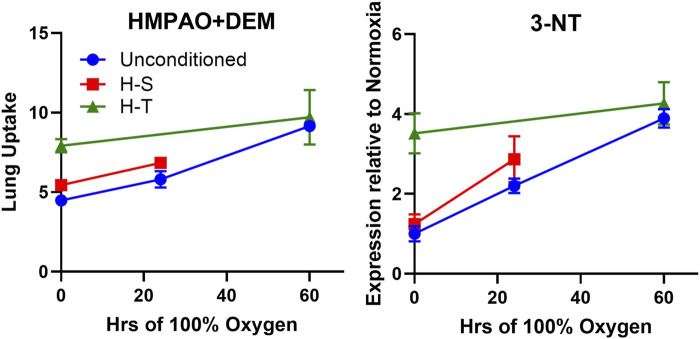
Independent markers of oxidoreductive state measured in unconditioned (blue circles), H-S (hyperoxia-sensitive, red squares), and H-T [hyperoxia-tolerant, green triangles (Audi et al., 2022b)] rats exposed to up to 60 h of >95% O_2_ hyperoxia. Left: DEM-insensitive ^99m^Tc-HMPAO (GSH-independent signal), Right: 3-NT expression normalized to unconditioned value.

Figure 7 right shows correlation between 3-NT expression, a marker of oxidative stress induced by reactive nitrogen species, and the DEM-insensitive component of ^99m^Tc-HMPAO lung uptake. Furthermore, Figure 10 shows that changes in the DEM-insensitive portion of ^99m^Tc-HMPAO uptake (left) in unconditioned, H-S, and H-T rats in response to hyperoxia exposure mirror the corresponding changes in lung tissue 3-NT expression (right).

Additional studies would be needed to assess the contribution of mitochondrial and 3-NT related processes to the DEM-insensitive portion of 99mTc-HMPAO lung uptake.

### ^99m^Tc-duramycin lung uptake

**FIGURE 11:**
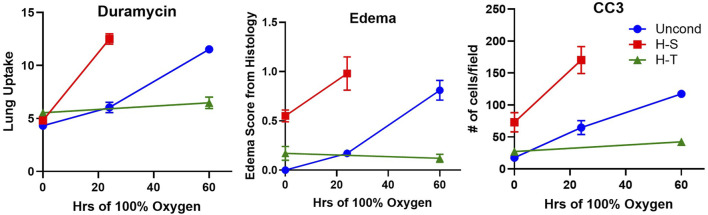
Independent markers of cell death measured in unconditioned (blue circles), H-S (hyperoxia-sensitive, red squares), and H-T [hyperoxia-tolerant, green triangles (Audi et al., 2022b)] rats exposed to up to 60 h of >95% O_2_ hyperoxia. Left: ^99m^Tc-duramycin, Center: histological edema score, Right: Number of cleaved caspase 3 (CC3) positive cells per field.

^99m^Tc-duramycin is a SPECT biomarker of tissue injury sensing cell death via apoptosis and/or necrosis (Audi S. et al., 2012; Audi et al., 2017; Audi et al. 2015; Audi et al. 2022b; Clough et al., 2012). Previously we demonstrated that lung uptake of ^99m^Tc-duramycin is highly correlated with the number of CC3-positive cells, and that apoptotic endothelial cells contribute more than other cell types to the enhanced ^99m^Tc-duramycin lung signal in hyperoxic rats (Audi et al., 2015). Data from the present study are consistent with this result (Figure 6 center) and show that changes in ^99m^Tc-duramycin lung uptake in unconditioned, H-T, and H-S rats in response to hyperoxia are similar to those of both CC3-positive cells and histological edema scores (Figure 11).

Results from previous studies indicated high correlation between apoptosis (as measured by ^99m^Tc-duramycin lung uptake and CC3 positive cells) and microvascular permeability lung injury (*K*_*f*_) (Gill et al., 2014; Martin, Nakamura, and Matute-Bello, 2003; Medhora et al., 2016). The higher edema score in lungs of H-S rats compared to unconditioned rats measured in the present study (Table 4) is consistent with the high *K*_*f*_ previously measured in unconditioned rat lungs (Table 5) (Taheri et al., 2024). The edema score was also significantly higher in lungs of H-S+24 rats than H-S rats (Table 4). In comparison, we have previously reported that this edema score did not increase in lungs of H-T rats following exposure to hyperoxia for 60 h (Table 5) (Audi et al., 2022b). Exposure to hyperoxia of both unconditioned and H-S rats decreased the expression of mitochondrial complex I (Figure 8).

Figure 6 (right) shows a strong relationship between CC3 and mitochondrial complex I expression. This is consistent with release of cytochrome c triggered by mitochondrial dysfunction, leading to apoptosis, caspase activation, and eventual cell death (Jiang and Wang, 2004). In combination with other data in this manuscript, our results provide new evidence that even moderate hyperoxia (FiO_2_ = 60%), associated with very few histological or systemic signs of damage, can predispose to lung injury in preclinical animal models, consistent with previous reports (Minkove et al., 2023; Hayatdavoudi et al., 1981).

### Limitations

One potential limitation of the present study is that the imaging and injury endpoints were not measured longitudinally in the same rats from pertinent groups (unconditioned vs. unconditioned+24 and H-S vs. H-S+24), but rather in independent groups of rats. (The animals were distributed over different order groups all from the same vendor.) Also, all rats used in this study were male in order to provide comparison of our results with previously reported data, since we are not aware of evidence that ARDS susceptibility (H-S) can be induced in female rats. Based on the promising results here, future studies could address these limitations. We also note that all results from rodents might not parallel exactly those in humans due to species differences, including that some rat species are burrowing animals and may experience periods of relative hypoxia and/or hypercarbia. Another limitation is that lung uptake of ^99m^Tc-HMPAO and ^99m^Tc-duramycin is affected predominantly by pulmonary endothelial cells since these cells are in direct contact with blood and account for ∼50% of the lung cells, whereas lung tissue assays, including glutathione tissue content and lung tissue expression of 3-NT, are affected by all lung cells. Nonetheless, good correlation with imaging quantification is demonstrated by the strong correlation in Figures 5, 6 (right).

### Potential implications of the imaging results

In a clinical setting, information about serial, directional changes in oxidative processes, whether derived from imaging or other diagnostic tests, could aid clinicians in stratifying the risk of ARDS progression in individual patients. Specifically, a patient exhibiting rising levels of ^99m^Tc-duramycin, alongside either increasing or stable ^99m^Tc-HMPAO lung uptake, may be at higher risk for oxidative lung injury compared to those showing stable or decreasing uptake of both ^99m^Tc-duramycin and ^99m^Tc-HMPAO. Individuals at elevated risk for oxygen-induced lung injury may be candidates for interventions such as tolerance of lower blood oxygenation (e.g., 86% saturation threshold), lower Hb thresholds for transfusions (due to the risk of transfusion reactions) or prone positioning. However, these therapies each carry risks of their own, and thus are not generally applied to all persons with ARDS. For example, switching a patient from the supine to prone position carries a risk of accidental extubation or inadvertent removal of central lines. This demonstrates the importance of tests such as SPECT imaging that can identify those with favorable risk/benefit ratios for interventions not universally implemented. At present*,* no reliable, noninvasive tests or scales to meet this need are available. For this reason, other markers of cell death and oxidative lung injury should be investigated. If their value is established, serial images using ^99m^Tc-HMPAO and ^99m^Tc-duramycin (or other readily acquired markers of cell death and/or oxidative stress) have the potential to enhance the value of existing lung injury prediction scores to better define risks for progression or improvement in *individual* patients with ARDS (Gajic et al., 2011; Daenen et al., 2025).

## Data Availability

The raw data supporting the conclusions of this article will be made available by the authors, without undue reservation.
