# Supplementary material for: Lung uptake of two SPECT markers identifies sensitivity to hyperoxia-induced acute respiratory distress syndrome in rats
Source: Front Physiol. 2025 Sep 12;16:1648159. doi: 10.3389/fphys.2025.1648159 (PMC12463966; doi:10.3389/fphys.2025.1648159)
Supplement: Supplementary file 1 [file Table1.docx]

**H&E Image Analysis**

**Step 1: Import H&E image files into *NDP.view2 (Hamamatsu)***

***ndpi*** images were opened using *NDP.view2*

**Step 2: Randomized Image Sampling**

Six randomly chosen regions per whole image at 20x magnification were selected on each slide.

**Step 3: Histological Injury Scoring**

Each image field was scored independently by trained investigators using visual inspection based on the histological injury endpoint scale presented in the manuscript.

**Step 4: Data Averaging and Scoring Summary**

The average score from the six sampled regions was determined for each of the three features (neutrophilic influx, edema, and alveolar thickness).

**CC3 Immunostained Image Analysis Using Thresholding**

**Step 1: Import the CC3 Image into Image J**

Open ImageJ → File → Open → Select image.

**Step 2: Convert to RGB Color Space**

Go to Image → Type → RGB Color

**Step 3: Launch Color Threshold Tool**

Go to Image → Adjust → Color Threshold → color space (RGB)

Select the threshold color (“Red”), thresholding method (“Default”)

**Step 4: Configure Channel Sliders (R, G, B)**

Red Channel (R): Set Minimum = 0, Maximum = 255

Green Channel (G): Set Minimum = 0, Maximum = 255

Blue Channel (B): Set Minimum = 0, Maximum = 140

**Step 5: Analyze Particles**

Go to Analyze → Analyze Particles

Output**: Count** = number of brown-stained particles per field
