# Supplementary figures and images for: Lung uptake of two SPECT markers identifies sensitivity to hyperoxia-induced acute respiratory distress syndrome in rats
Source: Front Physiol. 2025 Sep 12;16:1648159. doi: 10.3389/fphys.2025.1648159 (PMC12463966; doi:10.3389/fphys.2025.1648159)

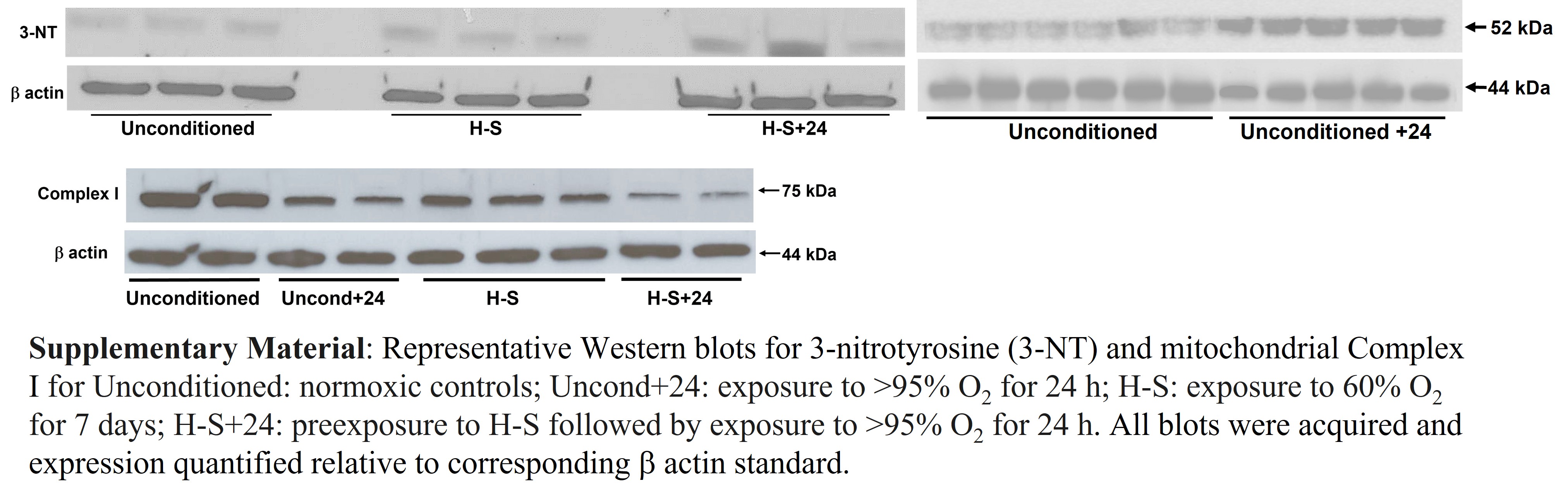

Supplement: Supplementary file 2 [file Image1.jpeg]
